# Effects of Cornelian Cherry on Atherosclerosis and Its Risk Factors

**DOI:** 10.1155/2019/2515270

**Published:** 2019-02-17

**Authors:** Jan Lietava, Nikoleta Beerova, Svetlana V. Klymenko, Elena Panghyova, Ivan Varga, Olga Pechanova

**Affiliations:** ^1^1st Department of Internal Medicine, Medical Faculty of Comenius University, Bratislava, Slovakia; ^2^Institute of Normal and Pathological Physiology, Centre of Experimental Medicine, Slovak Academy of Sciences, Bratislava, Slovakia; ^3^M.M. Gryshko National Botanical Garden of National Academy of Sciences of Ukraine, Kiev, Ukraine; ^4^Research Institute of Nutrition, Bratislava, Slovakia; ^5^Institute of Histology and Embryology, Medical Faculty of Comenius University, Bratislava, Slovakia

## Abstract

Functional food represents an important alternative management of atherosclerosis, its risk factors, and clinical complications. Atherosclerosis is characterized by microinflammation, formation of atheromatous lipoprotein-rich plaques, and protrombogenic status. Cornelian cherry (*Cornus mas L*., CC) contains polyphenols influencing all three components of atherosclerosis. Its high antioxidant potential, verified in experimental studies, exhibited a pronounced decrease of inflammatory markers. CC treatment demonstrated a favourable effect on lipid spectrum (comparable with statins), decrease of glycemia, and increase of insulin (comparable with glibenclamide). Polyphenols identified in CC exhibited both direct antiplatelet effects and reduction of platelet hyper-reactivity mediated via attenuation of oxidative stress. The first clinical trials confirmed a clinically relevant decrease of total and low-density lipoprotein cholesterol, triacylglycerols, lipoproteins, amelioration of inflammatory activity, and insulin secretion improvement after the treatment with CC polyphenolic compounds. However, the limitation of published studies is the use of undefined cultivars of CC, their experimental nature, small scale, and missing longitudinal trials. Nevertheless, biochemical properties of CC, hitherto described, predispose its products for the adjuvant management of atherosclerosis.

## 1. Introduction

Cornelian cherry (*Cornus mas L*., CC) is used as a food item as well as a traditional herbal drug in wide belt from middle and south Europe, through the Asia Minor and Caucasus to the Sub-Himalayan region. The archeological evidence of its use is dated to the La Tène period (range ca 450 B.D. to 100 A.D.) [1]. A Traditional medicine practice utilizes its anti-inflammatory and hypoglycemic properties in therapy of fever, dyspepsia and diabetes mellitus, rheumatic pain, nonspecific skin and urinary tract infections, and liver diseases in either a form of over-the-counter (OTC) herbal medical products or natural fruits and leaves [2–4]. The fruit of *C. mas*, together with the fruit of *C. officinalis*, has also a long history of use in traditional Chinese medicine. Known as *shānzhūyú*, it is used to retain the jing, essence, to tonify the kidneys, and in cases of spermatorrhea [5].

Beneficial effects of CC result from its favourable properties, mainly (i) fresh cornelian cherry fruits contains twice as much ascorbic acid as oranges; (ii) compared to other juices obtained from plum, pear and apple, cornelian cherry juice contains high levels of calcium, reaching 10 folds higher than other juices; (iii) CC has high contents of potassium and magnesium but is low in sodium and other essential minerals like Cu, Mn, Fe, and Zn; (iv) CC levels of toxic elements are also negligible; and (v) the fruits are excessively rich in organic acids, tannins, anthocyanins, phenols, and other antioxidants [6–9].

The first scientific analysis reported the CC content of anthocyanins in 1939 [10] confirmed long-term research on active compounds of CC. The crude extracts of fruits and other parts of the plant as well as their pure isolates reach in polyphenolic compounds exhibit a broad spectrum of pharmacological activities such as antimicrobial, antidiabetic, antiatherosclerotic, antiparasitic, anticancer, cytoprotective, hepatoprotective, neuroprotective, and renal-protective, antiplatelet, and antiglaucomic activities [11, 12].

Interest in new sources of anti-inflammatory and antioxidant compounds has recently become a major research issue. Cornelian cherry receiving particular attention for its significant amounts of phenolic compounds and vitamins, which exhibit a wide range of biological and pharmacological properties, may represent a promising source capable of fighting against atherosclerosis. This study was aimed at increasing knowledge regarding the effect of Cornelian cherry on an atherosclerotic process.

## 2. Atherosclerotic Process

Atherosclerosis, its clinical complications, and associated diseases such as myocardial infarction, stroke, diabetes mellitus, peripheral arterial occlusive disease, and hypertension are the leading causes of total mortality in industrial countries [13]. The detailed recognition of the etiopathology provides a background for the targeted therapy.

Life lasting microinflammation has a crucial role in the pathogenesis of atherosclerosis and subsequent vascular damage [14, 15]. The complex atherosclerotic process is generalized in humans independently of age [16], and it is accelerated by risk factors such as dyslipidemia, hyperglycemia and/or diabetes mellitus, obesity and/or unbalanced nutrition, hypertension, sedentarism, smoking, permanent stress, and environmental factors [17]. Atherosclerosis is in short characterized by excessive fibrosis of the intima, fatty plaque formation due to accumulation of predominantly low-density lipoprotein (LDL) cholesterol, proliferation of smooth muscle cells due to increased oxidative stress and/or cytokines, and infiltration and/or migration of a group of cells such as monocytes, T cells, and platelets, which are formed in response to progressing microinflammation [18]. Penetration of the cells into the vascular wall is conditioned by the expression of leukocyte and chemokine adhesion molecules in which the transcription is performed by nuclear factor-*κ*B (NF-*κ*B). Proinflammatory molecules, such as tumor necrosis factor *α* (TNF-*α*), interleukin 6 (IL-6) and interleukin 18 (IL-18) cytokines, C-reactive protein (CRP), adhesion molecules, matrix metalloproteinases (MMP-9 or gelatinase B) produced by monocytes, macrophages and/or adipose tissue further potentiate microinflammation, and oxidative stress [19, 20].

Inhibitory monocyte chemokine protein (MCP-1) increased level is early and persistent markers of progression of the process. Another important consequence of endothelial damage is the dysfunction of endothelial nitric oxide synthase (eNOS)/NO pathway with the decrease of eNOS or NO levels and impairment of a natural antiproliferating and anti-inflammatory effect of NO [21–24].

Atherosclerosis accelerates by an increased oxidation of LDL cholesterol to oxLDL cholesterol and/or by hyperglycemia [25, 26]. Severe damage to the arterial wall is associated with increased secretion of cytokines and growth factors as interleukin 1 (IL-1) and tumor necrotising factors by smooth muscles and endothelial cells, which causes penetration of various cells into a plaque. The core of a plaque consists of debris from cell lesions, foam cells, calcium, cholesterol esters, and a mass of fatty substances [27]. Their exposition to the blood initiates coagulation cascade in a vulnerable plaque [28]. An atherosclerotic process itself is associated with an increased level of fibrinogen, acute-phase reactant responding to inflammations or infections, and with increased level of factor VII, coagulate protein involved in thrombogenesis [29, 30].

## 3. Active Antiatherosclerotic Compounds in Cornelian Cherry

The CC fruit content varied dependently on the region and genotype in the range of 0.1–0.3% fat, 0.4% protein, 21.7% carbohydrate, 0.8% ash, 0.5% dietary fibre, 6.6–25.2% total sugar, (33.1–43.1% fructose and 53.6–63.1% glucose), 4.22–23.01% reducing sugars, and 14.96–38.87 mg/100 g of vitamin C, as well as 15 amino acids [31–34].

CC juice contains high level of calcium (323 mg/L) exceeding 10-fold content in plum, pear, or apple juices and a comparable amount of potassium, sodium, zinc, and magnesium, while copper level is significantly lower [9].

CC fruit has been found to contain a wide range of phytochemicals with a biological effect, including tannins (131.51–601.2 mg/L), organic acids (4.6–7.4%), anthocyanin, fatty acids, flavonoids [35], and at least 16 phenolic compounds which generally vary from 29.76 to 74.83 mg/g dry matter [36]. The content of CC anthocyanins varied between 36.35 and 116.38 mg/100 g and consists of delphinidin 3-O-beta-galactopyranoside, cyanidin 3-O-beta-galactopyranoside, and pelargonidin 3-O-beta-galactopyranoside, myricetin-3-arabinose, quercetin-3-galactoside, and gallic acid [32, 36, 37]. Of special interest is the high level of polyphenolic compound gallic acid (45.5 mg/g) with highly expressed antioxidant activity evaluated as a ferric-reducing antioxidant power (FRAP), which was 10-fold higher in CC in comparison with apple, pear, or plum [6, 9]. Sochor et al. [38] identified a chlorogenic acid in all assayed samples of Eastern and Middle Europe CC cultivars (Nero, Titus, and Vygotsky) as the major polyphenolic compounds. In contrast, Pawlowska et al. [39] found a quercetin 3-O-D-glucuronide as the major flavonoid constituent of CC fruits from the Italian region. In South-East Serbia CC genotypes, high level of (+) catechin and (–) epicatechin (41.23% and 41.96%, respectively) together with lower content of procyanidin B2 (16.80%) has been found [40].

In addition, terpene and other secondary metabolites [41] may importantly contribute to high antioxidant capacity and beneficial properties of different CC cultivars. Although the terpenes with proven antioxidant properties like *α*-phellandrene and *β*-myrcene were found in different investigated fruits, the highest content of these compounds was measured in cherry silver berry fruits [42]. Since secondary metabolites represent an integrated defence mechanisms of the plants, such properties may reflect beneficial effects on the health [43]. Due to strong antioxidant and anti-inflammatory properties, all polyphenolic compounds mentioned above have a big chance to fight against all phases of the atherosclerotic process.

Similarly, different polyphenolic compounds may significantly contribute to the nutraceutical effect of plants such as grapevine [44, 45], *Aronia melanocarpa* [46] and many others.

## 4. Effects of Cornelian Cherry on Atherosclerosis

### 4.1. “*In Vitro*” and Animal Studies

CC is capable to positively influence the classical risk factors of atherosclerosis. There is important evidence for the mutual antioxidant and hypolipidemic effect of CC, which could be mediated mainly by an antioxidant-related effect shown both *in vitro* and *in vivo* experimental conditions.

Sozański et al. [47] studied effects of CC fruit lyophilisate on peroxisome proliferator-activated receptor *α* (PPAR *α*) protein expression and atheromatous plaque changes in hypercholesterolemic rabbits. CC in a dose of 100 mg/kg BW caused a 44% decrease in triacylglycerols (TG) and prevented the development of atheromatous changes in the aorta. Amelioration of atherosclerosis was associated with a significant elevation of hepatic PPAR *α* protein expression which was considered a central mechanism protecting against fibrinogenesis and neoformation of collagen [48]. An antiatherogenic effect of CC on hepatic function was supported also by Celep et al. [49] who observed increased total antioxidant capacity of the liver, however, without changes in the activity of antioxidant enzymes like superoxide dismutase (SOD), catalase, glutathione peroxidase (GPx), and hepatic lipid peroxidation.

Favourable hypolipidemic effects of CC were found by Asgary et al. [50] in rats with alloxan-induced diabetes. The rats exhibited also an antihyperglycemic effect comparable with glibenclamide therapy in the control group. Rasoulian et al. [51] confirmed the hypoglycemic effect of CC in hamsters fed with CC fruits with subsequent elevation of insulin level partially independent on the dose. Preventive use of CC fruits two or three times daily did not provide any additional benefit in comparison with one dose. According to the experimental study using an immortalized proximal tubule epithelial cell line, anthocyanins, to which CC are rich, decrease a high-glucose-induced enhance cholesterol efflux and ATP-binding cassette transporter (ABCA1) expression [52]. Furthermore, anthocyanins cause an increase of PPAR *α* and liver X receptor *α* expression and a decrease of the high-glucose-induced expression of the proinflammatory cytokines, intercellular adhesion molecule-1 (ICAM-1), MCP1, and transforming growth factor-*β*1 (TGF-*β*1), as well as NF-*κ*B activation [52], which may explain the favourable effect of anthocyanins on blocking cholesterol deposition and their beneficial anti-inflammatory action.

Asgary et al. [53] compared the effect of 1 g/kg BW of CC powder with 100 mg/kg BW of lovastatin on fibrinogen levels in hypercholesterolemic rabbits. The study proved significant reduction of fibrinogen levels in both arms; in lovastatin one demonstrated a decrease of about 30 mg/dL, and in CC arm a decrease of about 50 mg/dL revealing more profound anti-inflammatory property of CC. Another study of Asgary et al. [50] in alloxan-induced diabetic rats demonstrated a beneficial effect on lipid spectrum, protecting the worsening of the spectrum in the CC-treated group. Generally, the hypolipidemic effect in animal studies corresponds to that induced by less potential statins. In hypercholesterolemic rabbits, Sozański et al. [54] found a decrease of total and LDL cholesterol, oxidised cholesterol, and TG as well as an increase of HDL cholesterol and hepatic PPARS *α* and *γ* after 60 days of therapy with anthocyanins derived from CC.

Protective effects of CC on endothelial function are much less studied despite predisposing favourable properties of the plant. The only relevant histological observation was made by Sozański et al. [54] in the group of cholesterol-fed rabbits, in which CC-derived anthocyanins significantly improved the composition of the arterial vessel, wall increasing intima thickness, and intima/media ratio in the thoracic aorta.

Leskovac et al. [55] demonstrated reduced incidence of radiation-induced micronuclei (19.23%) and reduced lipid peroxidation (50.04%) and two-fold enhancement of cell apoptosis in human peripheral blood lymphocyte cell cultures treated by an extract from the CC leaves. An analogous protective effect was found in Wistar rats treated with freeze-drying lyophilisate CC powder and exposed to high-fat or fructose diets. CC caused the decrease of the plasmatic while the increase in brain catalase activity suggesting increased cerebral protection. In turn, with regard to paraoxonase activity in both brain tissue and plasma, it had a stimulating effect. [56]. In the kidney injury induced by an injection of carbon tetrachloride, Banihabib et al. [8] observed the decrease of antioxidant enzyme activities (SOD, catalase, and GPx) and impairment of renal functions (increased serum creatinine, urea, and uric acid and decreased albumin). Treatment of rats with different doses of CC fruit extract (300 and 700 mg/kg) significantly ameliorated the alterations induced with carbon tetrachloride in lipid peroxidation, antioxidant defences, and biochemical and renal lesions.

Study of Asgary et al. [53] demonstrated strong attenuation of fibrinogen production after short-term therapy by CC extracts; however, studies of CC direct effect on atherothrombogenesis are missing. Another study of Williams et al. [57] using purified anthocyanins revealed reduction of expression of P-selectin suggesting an ability of anthocyanins to reduce thrombocyte activation. Authors supposed a potential contra-reaction of anthocyanins to oxidative stress-induced dysfunction of platelets. Yang et al. [58] confirmed a significant inhibition of platelet aggregation in the reduction of thrombus growth in human and murine blood using delphinidin-3-glucoside, which is also an important component of CC anthocyanins.

### 4.2. Human Studies

In the only two PubMed published controlled clinical studies, Asgary et al. [59] studied the hypolipidemic and anti-inflammatory effect of 100 grams of CC fruits added to a diet for 6 weeks in 40 dyslipidemic children and adolescent aged 9-16 years. The intervention group demonstrated a significant decrease of total cholesterol, TG, LDL cholesterol, apoB, ICAM-1, and VCAM-1 levels after six weeks. However, only apoA1 and ICAM-1 were significantly decreased compared to control groups. Clear improvement of lipid spectrum and inflammation markers after mild intake of CC added to usual diet predisposes the fruits as a supportive therapy of the main risk factors of atherosclerosis.

A randomized double-blind placebo-controlled clinical trial of Soltani et al. [60] on 60 patients with type 2 diabetes randomly was divided into two groups, treated either with 150 mg of anthocyanins or with placebo measured fasting plasma levels of glucose, insulin, HgbA_1C_, and TG as well as 2-hour postprandial glucose. After 6 weeks of intervention, an increase in insulin level (1.13 ± 1.90 *μ*U/mL versus −0.643 ± 1.82 *μ*U/mL) and a decrease in HgbA_1C_ (−0.24 ± 0.429% versus 0.023 ± 0.225%) and TG (−23.66 ± 55.40 mg/dL versus 2.83 ± 15.71 mg/dL) were observed. The authors concluded that daily consumption of the fruit extract of CC improves glycemic control by increasing insulin level and reduces TG serum level in type 2 diabetic adult patients.

**Figure 1:**
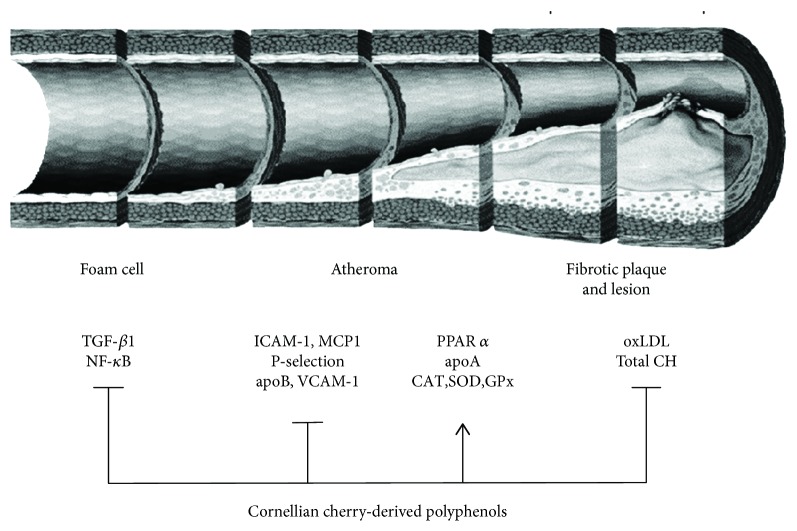
Effects of Cornelian cherry- (CC-) derived polyphenols on the atherosclerotic process. CC-derived polyphenols lead to the decrease of transforming growth factor-*β*1 (TGF-*β*1), nuclear factor-*κ*B (NF-*κ*B), intercellular adhesion molecule-1 (ICAM-1), monocyte chemokine protein 1 (MCP1), P-selectin, apolipoprotein B (apoB), vascular cell adhesion protein 1 (VCAM-1), oxidized low-density lipoprotein (oxLDL), and total cholesterol (CH) and the increase of peroxisome proliferator-activated receptor *α* (PPAR *α*), apolipoprotein A, catalase (CAT), superoxide dismutase (SOD), and glutathione peroxidase (GPx).

The effects of CC on atherogenetic, inflammatory, and antioxidant markers in experimental as well as clinical studies are summarized in Table 1. It seems that CC similar to other nutraceuticals reach in polyphenolic compounds [61, 62] may have beneficial effects on cardiovascular, diabetes, and obesity-related diseases including atherosclerosis (Figure 1). However, more studies on mechanisms, actions, pharmacokinetics, as well as adverse effects of CC extracts, their bioactive constituents, and their effective doses in humans are needed for the applications of CC extracts in clinical practice and treatment of atherosclerosis especially.

## 5. Conclusions

Cornelian cherry similar to other nutraceuticals is capable of favourable interaction with risk factors of atherosclerosis and can contribute to the prevention of atherosclerosis through a positive effect on lipid spectrum and glycemia, reduction of free radicals and inflammation, amelioration of endothelial dysfunction, and prothrombogenic status. Until now, an identification of the main responsible compound, dosing, and duration of therapy as well as clinical experience in prospective studies are missing.

## Figures and Tables

**Table 1:** Review of studies on effects of Cornelian cherry on atherogenetic, inflammatory, and antioxidant markers.

Authors	Study group(s)	Cornelian cherry	Duration	Dose	Parameters and results
Sozański et al. [47]	Hyper CH rabbits	Fruit lyophysate	60 d	100 mg/kg BW	↑ PPAR expression ↓ TG 44% ↓ Ao plaque ↓ ROS

Francik et al. [56]	22-week-old Wistar rats	Freeze-dried wild fruits	5 w	10% of daily intake	In brain tissue ↑ catalase ↑ PON1 ↑ FRAP ↓ protein carbonyl ↓ thiols

Banihabib et al. [8]	Wistar male rats, injected	Wild fruits, dried, methanol extracted powder	16 d	300 and 700 mg/kg BW	Kidney ↓ MDA ↑ CAT ↑ SOD ↑ GPx ↓ lipid peroxidation

Abdollahi et al. [7]	Wistar male rats	Hydromethanolic extract	21 d	50, 200, 400 mg/kg BW	Blood count improved in 400 mg group

Alavian et al. [63]	Wistar male rats, injected with CCl_4_	Wild fruits, dried, methanol extracted powder	16 d	300 res. 700 mg/kg BW	↓ hepatic markers AST, ALT, ALP ↓ hepatic lipid peroxidation

Asgary et al. [50]	DM rats (nonspecified)	Wild fruits, dried powdered	4 w	2 g/d	↓ G, TCH, LDL-CH, AST, ALT, ALP comparable to glibenclamide

Celep et al. [49]	Sprague–Dawley male rats	Leaves—80% methanolic extract	21 d	500 mg/kg BW	*In vitro*—free radical scavenging+metal reducing activity *In vivo*—↑ total antioxidative capacity of liver ↔ SOD, CAT, GPx, lipid peroxidation

Asgary et al. [59]	Dyslipidemic children 9-16 yrs	Wild fruits, fresh	6 w	100 gr fruits/d	↓ TCH, LDL-CH, TG, apoB, I-CAM1, VCAM-1 ↑ HDL-CH, apoA

Celep et al. [64]	Control rats vs. rats treated with CCl_4_	80% methanolic extract of CC leaves	21 d	100, 200, 500 mg/kg BW	Partial return of SOD, CAT, GPx, MDA, TEAC (depending on the dose)

Rasoulian et al. 2012 [51]	Hamsters	Fruits	20 d	5, 10, 15 g/d	↑ insulin ↓ body weight, ↓ G (only in 5 g/d)

Forman et al. [65]	Human breast cancer cells (MCF-7)	Aqueous leaf extracts	24, 48, 72 h	50-750 *μ*g/mL	Antiproliferative effects

Savikin et al. [66]	*In vitro* HeLa cells, LS174 cells of human cancer	Fruits and leaves	NA	Not specified	Direct correlation with antioxidant capacity

Yousefi et al. [67]	*In vitro* tumor cells	Hydroalcoholic CC extract	NA	5, 20, 100, 250, 500, 1000 *μ*g/mL	Inhibition of proliferation of different tumor cells in a dose-independent manner

Leskovac et al. [55]	*In vitro* human lymphocytes irradiated by gamma-rays	Wild leaves air-dried, powdered	NA	Extracts 0.1-0.4 mg/mL	Lowest dose—best results ↓ radiation-induced micronuclei ↓ lipid peroxidation

Jayaprakasam et al. [68]	C57BL mice	Fruits derived and purified anthocyanin vs. ursolic acid	8 w	1 g/kg of anthocyani-ns and 500 mg/kg of ursolic acid	Anthocyanine mice ↓ 27% weight gain ↓ TG ↓ lipid liver accumulation Both ↑↑ insulin

Ao, aortic; apoA, apolipoprotein A; apoB, apolipoprotein B; ALT, alanine aminotransferase; ALP, alkaline phosphatase; AST, aspartate aminotransferase; BW, body weight; CAT, catalase; CC, cornelian cherry; DM, diabetes mellitus; FRAP, ferric-reducing antioxidant power; G, glycemia; GPx, glutathione peroxidase; HDL-CH, high-density lipoprotein cholesterol; ICAM-1, intercellular adhesion molecule 1; MDA, malondialdehyde; LDL-CH, low-density lipoprotein cholesterol; PON, paraoxonase 1; PPAR, peroxisome proliferator-activated receptor; ROS, reactive oxygen species; SOD, superoxide dismutase; TCH, total cholesterol; TEAC, trolox equivalent antioxidant capacity; TG, triacylglycerols; VCAM-1, vascular cell adhesion protein 1.
